# *Polygonatum kiangnanense* (Asparagaceae), a new species from southeastern China

**DOI:** 10.3897/phytokeys.277.198454

**Published:** 2026-07-21

**Authors:** Tao Xu, Xiang-wen Song, Wei Wang, Wan-ting Li, Bang-xing Han, Shan-yong Yi

**Affiliations:** 1 Traditional Chinese Medicine Institute of Anhui Dabie Mountain, West Anhui University, Lu'an, 237012, Anhui, China Anhui Engineering Research Center for Eco-agriculture of Traditional Chinese Medicine, West Anhui University Luʼan China; 2 Anhui Engineering Research Center for Eco-agriculture of Traditional Chinese Medicine, West Anhui University, Lu'an, 237012, Anhui, China Traditional Chinese Medicine Institute of Anhui Dabie Mountain, West Anhui University Luʼan China; 3 State Key Laboratory for Quality Ensurance and Sustainable Use of Dao-di Herbs, Beijing, 100700, China State Key Laboratory for Quality Ensurance and Sustainable Use of Dao-di Herbs Beijing China

**Keywords:** Asparagaceae, new species, *Polygonatum kiangnanense*

## Abstract

*Polygonatum kiangnanense* (Asparagaceae), a new species from southeastern China, a newly identified species native to southeastern China, is herein described and illustrated in detail. It bears the closest morphological affinity to *P. odoratum*, yet can be clearly differentiated by a suite of distinct traits: a terete rhizome measuring 9–14 mm in thickness, stem angled, oblong leaf blades (8.0–13.0 × 3.5–5.0 cm), racemose inflorescences typically bearing 1–2 (occasionally 3) flowers, yellowish-green campanulate-cylindrical perianths with a constricted mid-section, perianth lobes 6–8 mm in length (accounting for half of the total flower length), filaments adnate to the lower two-thirds of the perianth (12–14 mm long, smooth, with no apical saccate protrusions), and black berries (6–8 mm in diameter) containing 2–4 seeds. The complete chloroplast genome of the sampled individuals of this new species ranges from 154,571 to 154,593 bp in length. Phylogenetic analyses based on chloroplast genome sequences confirm that *P. kiangnanense* is genetically distinct from morphologically similar congeners.

## Introduction

The genus *Polygonatum* Mill. (Asparagaceae, Polygonateae) represents the most species-rich genus within its tribe, with a broad distribution spanning the northern temperate, northern subtropical, and frigid zones. The highest level of species diversity for this genus is concentrated in the region extending from the Himalayas to northern East Asia (Meng et al. 2014; Wang et al. 2016; Xia et al. 2022). Worldwide, more than 80 species of *Polygonatum* have been taxonomically recognized, with over 40 being endemic to China. This underpins China’s status as a key hub for the species’ distribution and genetic diversification (Yang et al. 2022). In Traditional Chinese Medicine, Polygonati Odorati Rhizoma, sourced from the dried rhizomes of select *Polygonatum* species, serves a dual role as both a medicinal herb and an edible product. It is valued for its therapeutic properties, including nourishing yin and moistening dryness, promoting fluid production to quench thirst (Chinese Pharmacopoeia Commission 2025). Additionally, more than 20 other *Polygonatum* species are utilized for medicinal purposes in various regional practices, underscoring the genus’ considerable medicinal and economic significance (Zhao et al. 2022).

Species delineation within *Polygonatum* primarily relies on morphological characteristics such as leaf arrangement, filament morphology, flower size, shape and color, and rhizome structure (Baker 1875; Chen and Tamura 2000; Tamura et al. 2008). With the advancement of molecular biological techniques, chloroplast genome sequencing has emerged as a powerful tool for taxonomic identification and phylogenetic analysis of *Polygonatum*. This approach provides robust evidence for resolving the taxonomic boundaries of closely related species (Floden and Schilling 2018; Xia et al. 2022; Song et al. 2026).

During field surveys of wild *Polygonatum* germplasm resources conducted in southeastern China (encompassing Anhui, Hubei, and Zhejiang provinces), we collected plant specimens initially identified as *P. odoratum* due to their similar rhizome morphology. However, detailed morphological observations revealed notable differences in aboveground traits: a plant height ranging from 40 to 62 cm, oblong leaf blades, yellowish-green campanulate-cylindrical flowers with a constricted mid-section, and perianth lobes accounting for half of the total flower length. To confirm the taxonomic status of this plant, we conducted comprehensive comparative morphological studies, chloroplast genome sequencing, and phylogenetic analyses. The cumulative evidence demonstrates that this taxon represents a previously unrecognized species, which we formally describe herein as *Polygonatum kiangnanense* sp. nov. Although *P. odoratum* is a species with high morphological variability, detailed morphological analyses reveal that *P. kiangnanense* possesses several distinct diagnostic features. These traits far exceed the natural morphological variation range of *P. odoratum*, thereby supporting the recognition of this taxon as a previously undescribed new species.

## Materials and methods

### Population sampling

**Figure 1.**
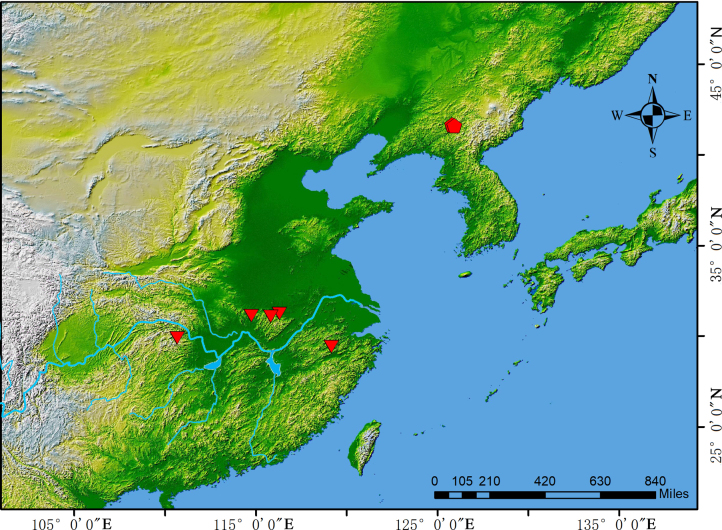
Distribution map of *Polygonatum kiangnanense* (red triangles) and *P. odoratum* (red pentagons) in China. The map was generated using ArcGIS 10.2 based on geographical coordinates of field collection sites. The blue lines represent the Yangtze River and its tributaries.

The putative new species was collected from five wild populations across Anhui, Hubei, and Zhejiang provinces during key developmental stages (growth initiation, flowering, and fruiting) between 2022 and 2025 (Fig. 1, Table 1). A total of five individuals were transplanted to the Botanical Garden of West Anhui University for long-term monitoring of morphological traits and phenological patterns. For comparative purposes, *P. odoratum* individuals were collected from Tonghua, Jilin Province, and cultivated under identical environmental conditions. Voucher specimens of all sampled individuals were deposited in the Herbarium of Anhui University of Traditional Chinese Medicine (ACM). Chloroplast DNA (cpDNA) sequences of the new species were generated through sequencing, while cpDNA sequences of other *Polygonatum* species (including *P. odoratum* and *P. infundiflorum* Y.S.Kim, B.U.Oh & C.G.Jang and outgroup taxa (*Heteropolygonatum alternicirrhosum* (Hand.-Mazz.) Floden and *H. ogisui* M.N.Tamura & J.M.Xu) were retrieved from the NCBI database.

**Table 1.** Information on the sampled and investigated populations.

**Species name**	**Collection site**	**Voucher number**	**GenBank number**
*Polygonatum kiangnanense*	Mazongling, Jinzhai, Lu’an city, Anhui Province, China	Xu JNYZ-1	PX404796
*P. kiangnanense*	Mazongling, Jinzhai, Luan city, Anhui Province, China	Xu JNYZ-2	
*P. kiangnanense*	Hong’an, Huanggang city, Hubei Province, China	Xu JNYZ-3	PX404797
*P. kiangnanense*	Wufenghouhe, Wufeng, Yichang city, Hubei Province, China	Xu JNYZ-4	
*P. kiangnanense*	Chun’an, Hangzhou city, Zhejiang Province, China	Xu JNYZ-5	
*P. odoratum*	Tonghua, Tonghua city, Jilin Province, China	Xu YZ-6	PX404798
*P. odoratum*	Tonghua, Tonghua city, Jilin Province, China	Xu YZ-7	

### Genome sequencing, assembly, and annotation

Genomic DNA was extracted from silica-gel-dried leaves of the new species using the Tiangen DNAsecure Plant Kit (DP320). Whole-genome sequencing was carried out on the BGISEQ–500 platform by Hefei Biodata Biotechnologies Inc. Raw sequencing reads were filtered using fastp (Chen et al. 2018), and the chloroplast genome was assembled with SPAdes version 3.10.0 (Bankevich et al. 2012). Genome annotation was performed using GeSeq (Tillich et al. 2017) and BLASTx (Gish and States 1993), and the plastid genome map was generated with OGDRAW (Greiner et al. 2019). The complete chloroplast genome sequences of *P. kiangnanense* (accession numbers: PX404796, PX404797) and the newly identified *P. odoratum* (accession number: PX404798) in this study have been deposited in GenBank. Basic characteristics of the chloroplast genomes of *P. kiangnanense*, *P. odoratum*, *P. falcatum*, *P. langyaense*, and *P. infundiflorum* were analyzed and compared using Geneious software (Markowitz et al. 2012) (Table 2).

**Table 2.** Basic characteristics of cpDNAs of *P. kiangnanense*, *P. odoratum*, *P. falcatum*, *P. langyaense*, and *P. infundiflorum*.

**Characteristic**	***P. kiangnanense***	***P. odoratum***	***P. falcatum***	***P. langyaense***	***P. infundiflorum***
Total length (bp)	154571–154593	155310	154,579	154578	154,578
GC%	37.7%–37.8%	37.8%	37.7%	37.7%	37.7%
LSC length (bp)	83,487–83,509	84237	83528	83527	83,527
SSC length (bp)	18460	18469	18457	18457	18457
IR length (bp)	26312	26302	26297	26297	26297
Total genes	133	133	133	133	133
Protein-coding genes	87	87	87	87	87
rRNA genes	8	8	8	8	8
tRNA genes	38	38	38	38	38

### Phylogenetic analysis

To clarify the phylogenetic position of the new species, a dataset was constructed comprising chloroplast genome sequences of *P. kiangnanense* (2 individuals), 48 *Polygonatum* species (67 individuals retrieved from NCBI), and 2 outgroup species (*H. alternicirrhosum* and *H. ogisui*). *Heteropolygonatum* is a genus closely related to *Polygonatum* within the tribe Polygonateae (Asparagaceae), sharing strong morphological affinities and a well-supported sister phylogenetic relationship. *H. alternicirrhosum* and *H. ogisui* are frequently employed as reliable outgroups for phylogenetic analyses in *Polygonatum* systematics, as confirmed by recent phylogenomic studies (Song et al. 2026). Notably, although numerous *P. odoratum* chloroplast genomes are available in GenBank, our preliminary phylogenetic analysis (Suppl. material 1) indicated that these sequences are polyphyletic. Most sequences clustering with *P. kiangnanense* should be misidentified specimens from southern China, whereas genuine *P. odoratum* from Northeast China forms a well-supported monophyletic clade clearly separated from *P. kiangnanense*. We therefore newly sampled and sequenced *P. odoratum* from Tonghua, Jilin (GenBank: PX404798) for robust phylogenetic inference. Maximum Likelihood (ML) analysis was performed using FastTree version 2.1.10 under the GTR + GAMMA substitution model. Node support was evaluated through 1,000 site-likelihood re-samplings and the Shimodaira–Hasegawa (SH) test (Price et al. 2010). Bayesian Inference (BI) analysis was conducted using MrBayes v3.2.7 (Ronquist et al. 2012) with the GTR+F+I+G4 model (selected via ModelFinder), and Markov chain Monte Carlo (MCMC) sampling was run for 100,000 generations.

## Results and discussion

**Figure 2.**
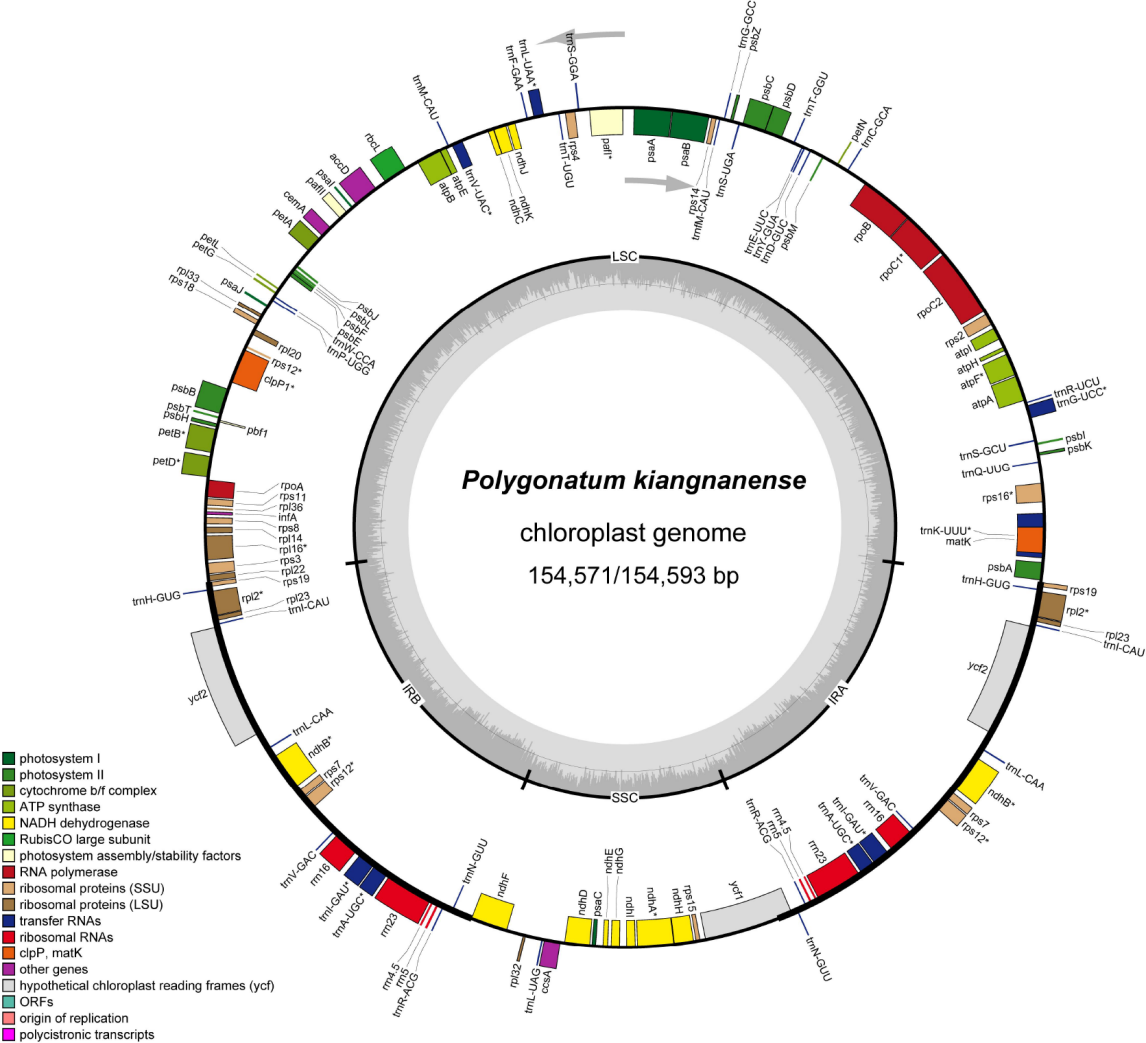
Plastid genome map of *Polygonatum kiangnanense* T.Xu, S.Y.Yi & X.W.Song, sp. nov.

The complete chloroplast genome of *P. kiangnanense* ranges from 154,571 to 154,593 bp in length, exhibiting the typical quadripartite structure characteristic of most angiosperms, consisting of a large single-copy (LSC) region, a small single-copy (SSC) region, and two inverted repeat (IR) regions (Fig. 2). The genome contains a total of 133 genes, including 87 protein-coding genes, 8 rRNA genes, and 38 tRNA genes, which is consistent with the gene content observed in other *Polygonatum* species (Xia et al. 2022; Song et al. 2026). Comparative analysis indicates that the chloroplast genome length of *P. kiangnanense* is shorter than that of *P. odoratum* (155,310 bp), with differences primarily localized to the LSC and SSC regions. A comprehensive summary of the distinctive features and statistical analyses of their cpDNAs is presented in Table 2.

**Figure 3.**
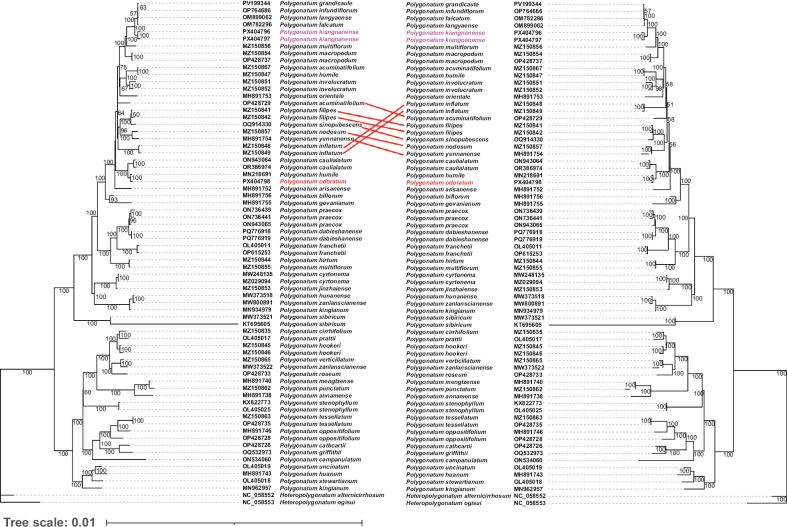
Phylogenetic analyses were performed to clarify the phylogenetic relationships between the newly described Polygonatum species and its closely allied taxa, using complete chloroplast genome sequences. The phylogenetic tree set comprises a maximum likelihood (ML) tree (left panel) and a Bayesian Inference (BI) tree (right panel), constructed based on 69 representative *Polygonatum* species, with Heteropolygonatum alternicirrhosum and Heteropolygonatum ogisui set as the outgroup taxa. Branch support values correspond to the bootstrap support scores from the ML analysis and the posterior probabilities from the BI analysis, respectively. The phylogenetic placement of *Polygonatum kiangnanense* is marked in pink, while that of *P. odoratum* is highlighted in red. The GenBank accession number of each taxon is annotated following the corresponding species binomial name.

Phylogenetic analyses (ML and BI trees) based on complete chloroplast genomes reveal that all individuals of *P. kiangnanense* form a well-supported monophyletic clade (bootstrap support [BS] = 100%, Bayesian posterior probability [BPP] = 100%; Fig. 3). This clade forms a sister relationship with the clade containing *P. falcatum*, *P. langyaense*, *P. infundiflorum*, and *P. grandicaule* with strong statistical support. Interestingly, *P. kiangnanense* did not cluster with *P. odoratum*, but rather showed a relatively greater phylogenetic distance from it. Despite the morphological similarity between *P. kiangnanense* and *P. odoratum* (Table 3), the significant genetic divergence and distinct morphological traits collectively confirm the status of *P. kiangnanense* as a new species. Furthermore, we have carefully checked the specimen records in the Chinese Virtual Herbarium (CVH) and found that, in addition to our collected specimens from Anhui, Hubei, and Zhejiang, some *Polygonatum* specimens deposited in CVH in recent years—previously identified as *P. odoratum*—actually represent *P. kiangnanense*. These specimens are documented from Anhui, Zhejiang, Hubei, and Hunan provinces, providing reliable evidence for predicting its potential distribution. Accordingly, *P. kiangnanense* is considered to be naturally distributed mainly in Anhui, Zhejiang, Hubei, and Hunan provinces within southeastern China.

**Table 3.** Comparison of *Polygonatum kiangnanense*, *P. odoratum*, and *P. infundiflorum*.

**Characters**	***P. kiangnanense***	***P. odoratum***	***P. infundiflorum***
Rhizome	terete, 9–14 mm thick	terete, 5–14 mm thick	terete, 7.5 mm thick
Stem	stem angled	Stem angled	Stem terete
Leaf (length × breadth)	8–13 cm × 3.5–5 cm	5–12 cm × 3–6 cm	11.2–14.4 cm × 3.4–6.6 cm
plant height	40–62 cm	20–50 cm	51–74 cm
flower (shape, color)	bell-shaped, mid-section constriction, yellowish green	bell-shaped, yellowish green to white	Funnel-shaped, mid-section constriction, yellowish green
segments of the perianth	6–10 mm	3 mm	6–8 mm
Anther	2.5 mm	4 mm	2.9 mm
Peduncle	5–25 mm	10–15 mm	10–15 mm
Distribution Area	Yangtze River Basin	widely distributed in the temperate regions of Eurasia	Endemic Species of Korea

### Taxonomic treatment

#### Polygonatum kiangnanense

Taxon classification

Plantae

AsparagalesAsparagaceae

T.Xu, S.Y.Yi & X.W.Song sp. nov.

896C0ED0-EFF7-5DEA-892F-2E7C45998E7B

urn:lsid:ipni.org:names:77387592-1

Figs 4, 5

##### Diagnosis.

*Polygonatum kiangnanense* shows affinities to *P. odoratum* and *P. infundiflorum* but can be differentiated from *P. odoratum* by its angulate stem (40–62 cm tall), oblong leaves, racemose inflorescences with fewer flowers (1–2(3) vs. 3–7(14)), yellowish-green campanulate-cylindrical perianths with a constricted mid-section (vs. straight tube), longer perianth lobes (6–8 mm vs. 3 mm), and fewer seeds per fruit (2–4 vs. 7–9). It differs from *P. infundiflorum* in its angulate stem (vs. terete), oblong leaves (vs. lanceolate), campanulate-cylindrical flowers (vs. funnel-shaped), and distribution in southeastern China (vs. Korean endemic).

**Figure 4.**
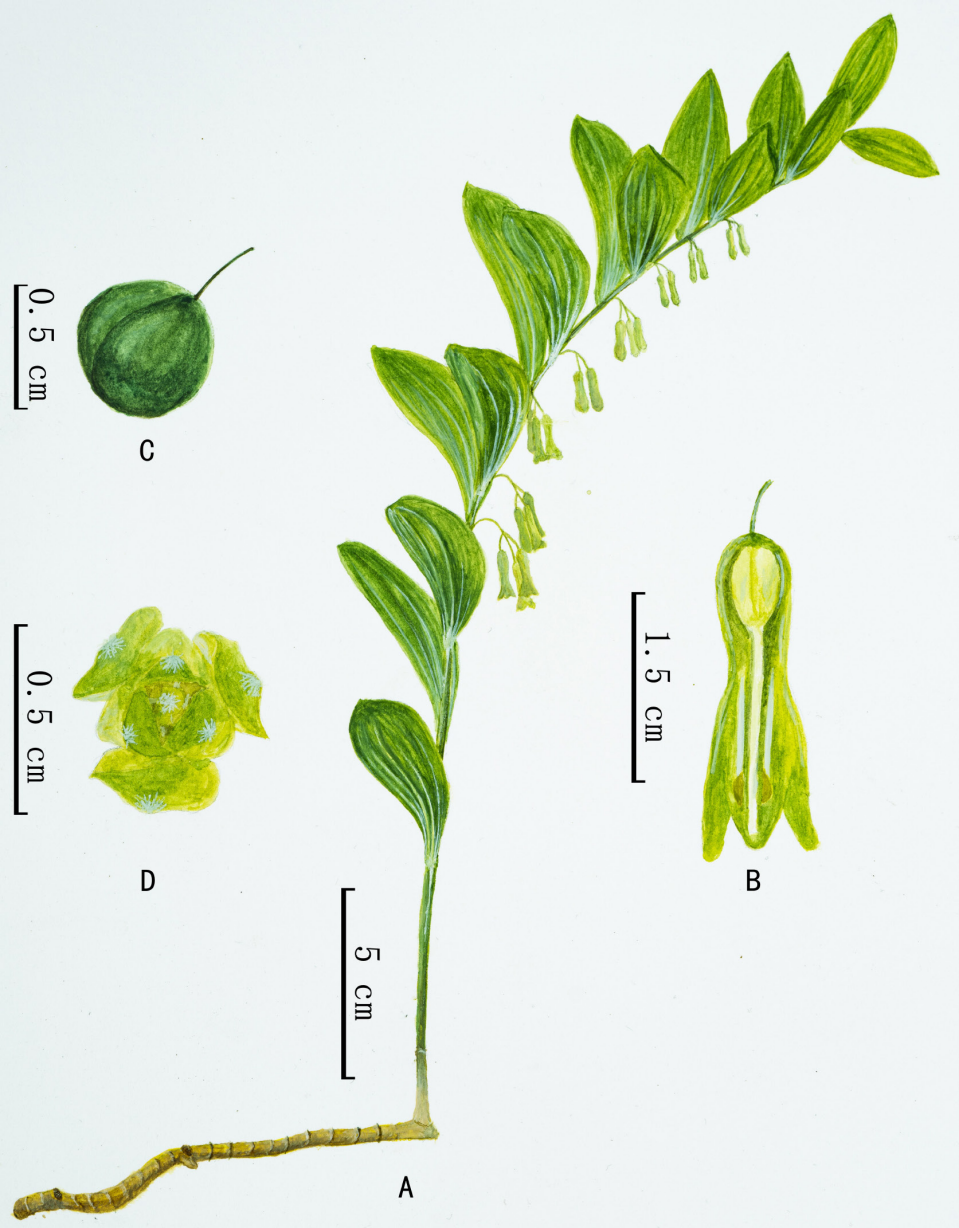
*Polygonatum kiangnanense* T.Xu, S.Y.Yi & X.W.Song, sp. nov. **A**. Plants; **B**. Longitudinal section of floral tube, showing stamens and pistil; **C**. Fruit; **D**. Flower. Drawn by Tao Xu.

**Figure 5.**
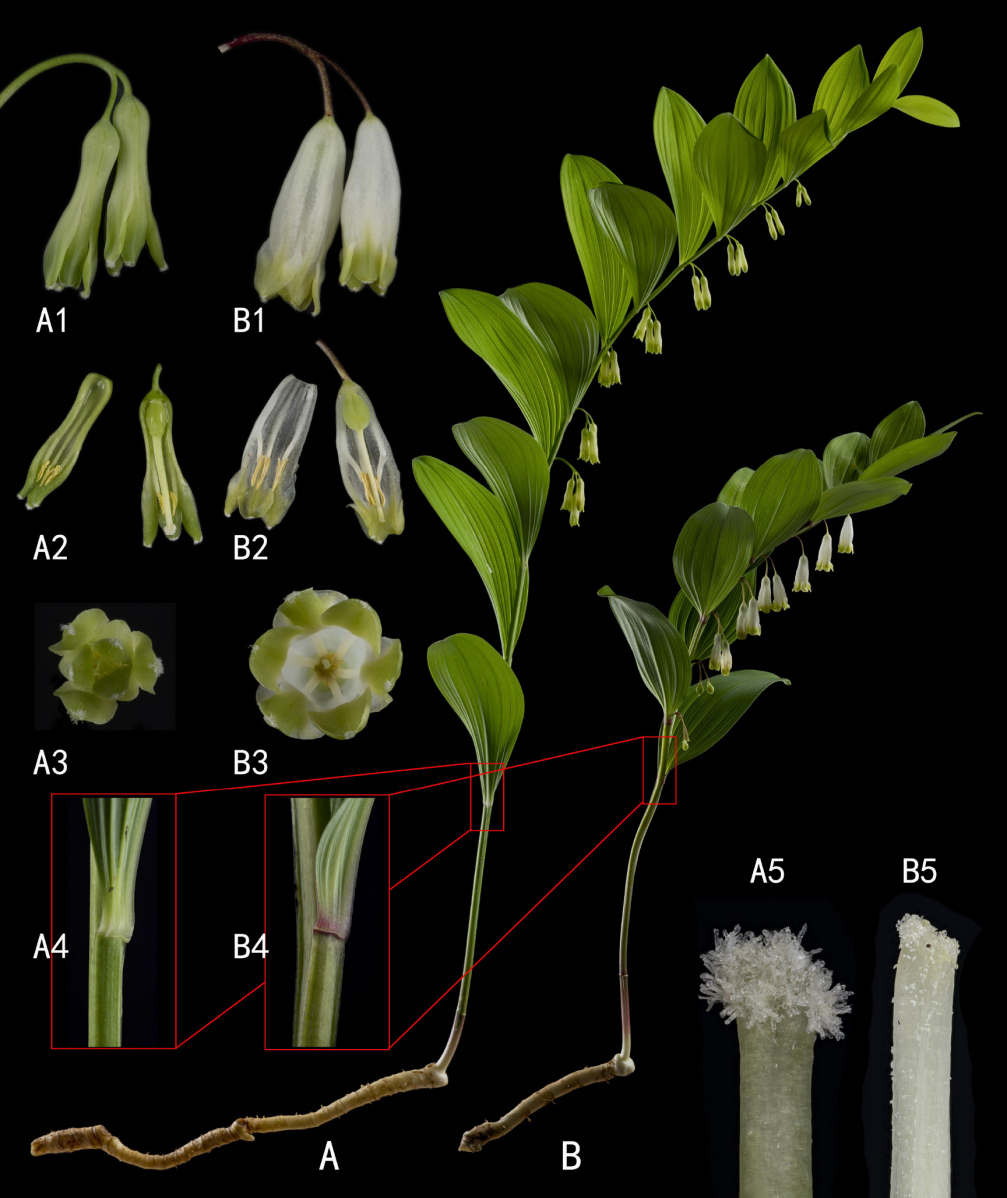
*Polygonatum kiangnanense* T.Xu, S.Y.Yi & X.W.Song, sp. nov. (**A**) and *P. odoratum* (**B**). **A1, B1**. Flower shape; **A2, B2**. Longitudinal section of floral tube, showing stamens and pistil; **A3, B3**. Flower; **A4, B4**. Stem ridge; **A5, B5**. Stigma. Photos by Tao Xu.

##### Type.

**Figure 6.**
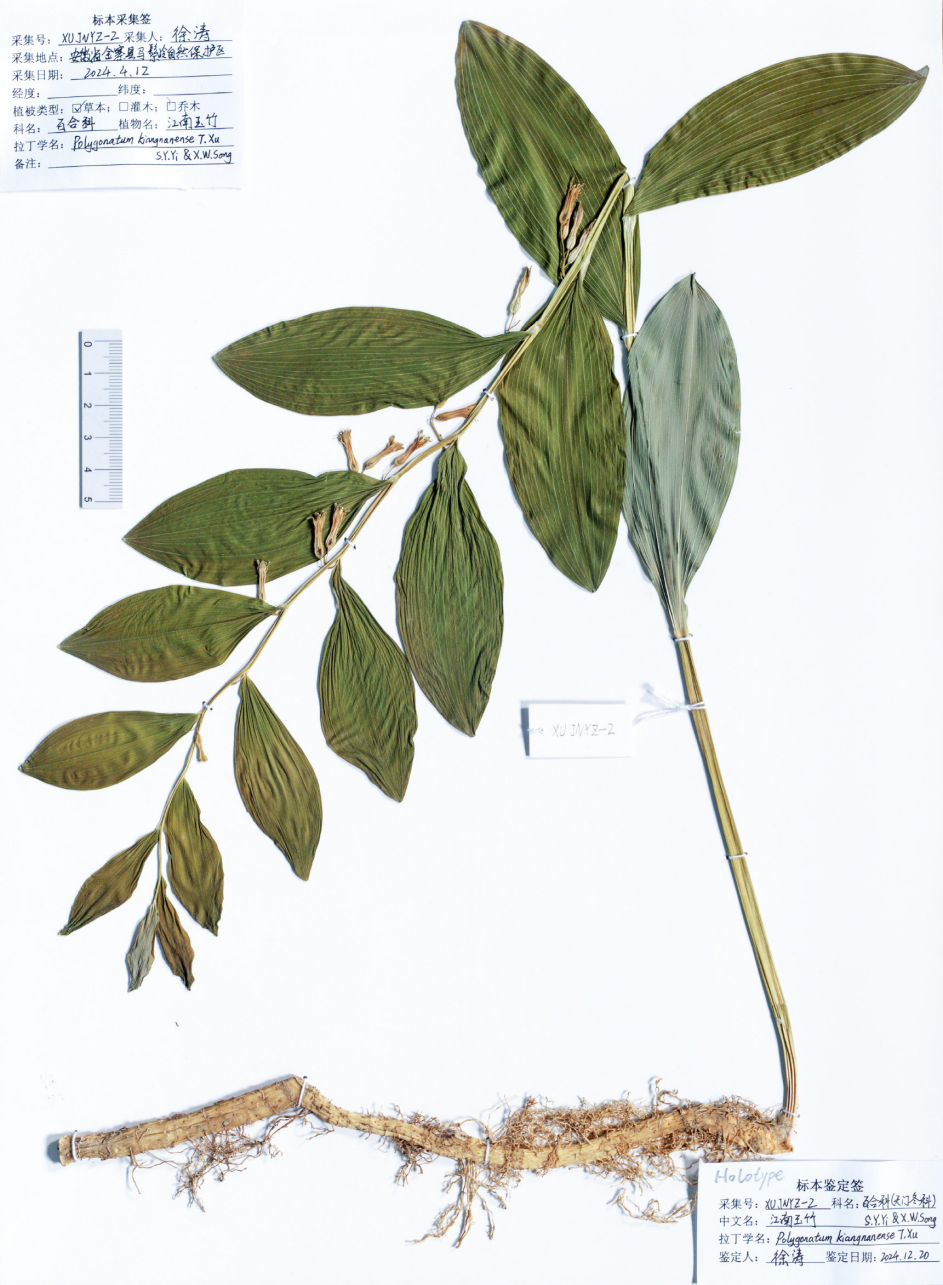
Holotype of *Polygonatum kiangnanense* T.Xu, S.Y.Yi & X.W.Song, sp. nov.

China • Anhui Province: Luan City, Jinzhai County, Mazongling Natural Conservation Zone, 1123 m.a.s.l., 12 April 2024, *Xu JNYZ-2*, (holotype: ACM, Fig. 6; isotype: ACM).

##### Description.

Herb, rhizome terete, 9–14 mm thick; transverse section white. Stem arching, green, 40–62 cm, glabrous, angled. Leaves 7–15, alternate; petiole short or nearly sessile; leaf blade oblong, 8.0–13.0 × 3.5–5.0 cm, erect; apex acuminate; base cuneate; veins prominently raised abaxially. Inflorescences numerous, racemose, borne in upper leaf axils, each with 1–2(3) pendulous flowers; Common peduncle 5–25 mm; pedicel 5–10 mm; Flowers grow in the axils of the leaves, pendulous. Perianth yellowish green, campanulate-cylindrical, mid-section constriction, 17–20 mm long; lobes 6–8 mm long, apex with filiform appendages. The lower 2/3 of the filament are connate with the perianth, cylindrical, 12–14 mm long, smooth, apex without saccate-convex. Anthers 2.5 mm long. Ovary 4 mm long, 2 mm in diam, style 10–13 mm long. Berries black, 6–8 mm in diam, 2–4 seeded.

##### Phenology.

Flowering March–April, fruiting April–October.

##### Distribution and habitat.

This species mainly grows under deciduous broad-leaved forests and bamboo forests (Suppl. material 2), 200–1300 m; documented on the slopes of South of the Huai River, with a notable concentration occurring south of the Yangtze River. Scatteredly distributed in the Dabie Mountains, in Anhui, Hubei and Zhejiang Provinces.

##### Etymology.

The specific epithet ‘kiangnanense’ refers to the Jiangnan region (south of the Yangtze River), where the species was originally discovered and where most of its populations occur.

##### Chinese name.

江南玉竹(jiang nan yu zhu).

### Morphological comparison

The new species is morphologically similar to *P. odoratum* in its cylindrical rhizomes, but it differs from *P. odoratum* in plant height (40–60 cm) (Figs 4, 5, Table 3). Total pedicel length, yellow-green flowers (vs. white or yellow-green flowers), narrow middle corolla (vs. straight corolla tube) and tepal length (6–8 mm). In morphology, this new species is obviously different from most species of *Polygonatum* through the thin cylindrical rhizome. The rhizome shape is similar to only *P. infundiflorum* and *P. odoratum*, but the stem of *P. infundiflorum* is cylindrical and has no edges and corners. The aboveground part of *P. odoratum* is greatly different from this new species and can be clearly distinguished.

### Identification key to the species of *Polygonatum* in China

**Table:** 

1a	Perianth (13–)15–30 mm long	**2a**
2a	Leaves alternate	**3a**
3a	Bracts leaf-like, ovate or lanceolate, 1–3.5 cm long, multi-nerved	**Ser. Bracteata**
3b	Bracts membranous or subherbaceous, subulate or linear-lanceolate, minute, rarely up to 1.2 cm long, nerveless or 3–5-nerved, or bracts absent	(**Ser. Alternifllia**)
4a	Rhizome cylindric	**5a**
5a	Perianth tube pubescent inside (at the point of filament attachment); filaments papillose to short-pilose	**6a**
6a	Plants relatively low, 20–30 cm high; rhizome 3–4 mm in diam.; leaves 4–5, 7–9 cm long; bracts minute, nerveless	***P. acuminatifolium***
6b	Plants relatively tall, 40–80 cm high; rhizome 6–14 mm in diam.; leaves 6–15	**7a**
7a	Plants with 6–9 leaves; petiole 5–15 mm long. Pedicel with a basal bract; perianth slightly constricted at mouth; lobes 2–3 mm long	***P. inflatum***
7b	Plants with 7–15 leaves; petiole short or nearly absent. Pedicel without bract; perianth constricted at middle; lobes 6–8 mm long	***P. kiangnanense***
5b	Perianth tube glabrous inside; filaments smooth to papillose; leaves sessile or with very short petioles	**8a**
8a	Leaf abaxial surface strigose	***P. humile***
8b	Leaf abaxial surface glabrous	**9a**
9a	Inflorescence with (3–)5–12(–17) flowers	***P. macropodum***
9b	Inflorescence with 1–2(–4) flowers	***P. odoratum***
4b	Rhizome zingiberaceous, moniliform, or somewhat moniliform	**10a**
10a	Pedicel with a basal bract equaling it in length (ca. 0.5 cm); anthers with a spur at apex	***P. franchetii***
10b	Pedicel without bract or with a minute bract; anthers without a spur at apex	**11a**
11a	Filaments filiform in upper part, slightly flattened in lower part, smooth	***P. arisanense***
11b	Filaments flattened on both sides throughout, papillose to short-villous	**12a**
12a	Leaf abaxial surface pubescent; peduncle slender, 3–8 cm long	***P. filipes***
12b	Leaf abaxial surface glabrous; peduncle relatively stout and short, 1–4 cm long	**13a**
13a	Plants relatively tall, 50–100 cm high; rhizome thick, 1–2 cm in diam.; leaves 10–15; inflorescence usually with 2–7 flowers	***P. cyrtonema***
13b	Plants relatively low, 15–40 cm high; rhizome slender, 5–7 mm in diam.; leaves 5–9; inflorescence with 1–2 flowers	***P. nodosum***
2b	Leaves verticillate or opposite	**14a**
14a	Plants tall, usually over 1 m high; leaves mostly verticillate, apex circinate; perianth adnate for at least 2/3 its length	**Ser. Kingiana**
14b	Plants dwarf, less than 10 cm high; leaves usually only ca. 10, often crowded together; flower solitary	**Ser. Hookeriana**
1b	Perianth 6–12(–15) mm long	**15a**
15a	Leaves mostly alternate, apex not circinate	**16a**
16a	Perianth united only at base (1–2 mm)	**Ser. Punctata**
16b	Perianth united into a urceolate tube	**Ser. Alte-lobata**
15b	Leaves mostly verticillate or opposite, rarely alternate	**17a**
17a	Leaves usually opposite; ovary 4–7 mm long; anthers 3–4 mm long	**Ser. Oppositifolia**
17b	Leaves usually verticillate; ovary 2–3 mm long; anthers 2–3 mm long	**Ser. Verticillata**

## Supplementary Material

XML Treatment for Polygonatum kiangnanense

## Additional information

### Artificial Intelligence (AI) use

The authors accept full responsibility for the content of the manuscript, including the disclosure of any use of AI.

No AI tools were used in the preparation of this manuscript.

### Author ORCIDs

T. Xu https://orcid.org/0009-0004-9155-3651

X.-w. Song https://orcid.org/0009-0001-3356-6428

W. Wang https://orcid.org/0000-0003-3100-6184

W.-t. Li https://orcid.org/0009-0003-0515-8317

B.-x. Han https://orcid.org/0000-0002-1696-6104

S.-y. Yi https://orcid.org/0000-0001-6653-1515

### Data availability

All of the data that support the findings of this study are available in the main text or NCBI (https://www.ncbi.nlm.nih.gov/).

## Supplementary materials

Supplementary material 1 A preliminary phylogenetic tree constructed with representative chloroplast genomes of all reported *Polygonatum* species Data type pdf https://binary.pensoft.net/file/1720937This dataset is made available under the Open Database License (http://opendatacommons.org/licenses/odbl/1.0/). The Open Database License (ODbL) is a license agreement intended to allow users to freely share, modify, and use this Dataset while maintaining this same freedom for others, provided that the original source and author(s) are credited. Tao Xu, Xiang-wen Song, Wei Wang, Wan-ting Li, Bang-xing Han, Shan-yong Yi

Supplementary material 2 *Polygonatum kiangnanense* habitat Data type pdf https://binary.pensoft.net/file/1720938This dataset is made available under the Open Database License (http://opendatacommons.org/licenses/odbl/1.0/). The Open Database License (ODbL) is a license agreement intended to allow users to freely share, modify, and use this Dataset while maintaining this same freedom for others, provided that the original source and author(s) are credited. Tao Xu, Xiang-wen Song, Wei Wang, Wan-ting Li, Bang-xing Han, Shan-yong Yi
