# Supplementary material for: Polygonatum kiangnanense (Asparagaceae), a new species from southeastern China
Source: PhytoKeys. 2026 Jul 21;277:227–40. doi: 10.3897/phytokeys.277.198454 (PMC13417040; doi:10.3897/phytokeys.277.198454)
Supplement: Supplementary material 1 — A preliminary phylogenetic tree constructed with representative chloroplast genomes of all reported Polygonatum species [file phytokeys-277-227_article-198454__-s001.pdf]

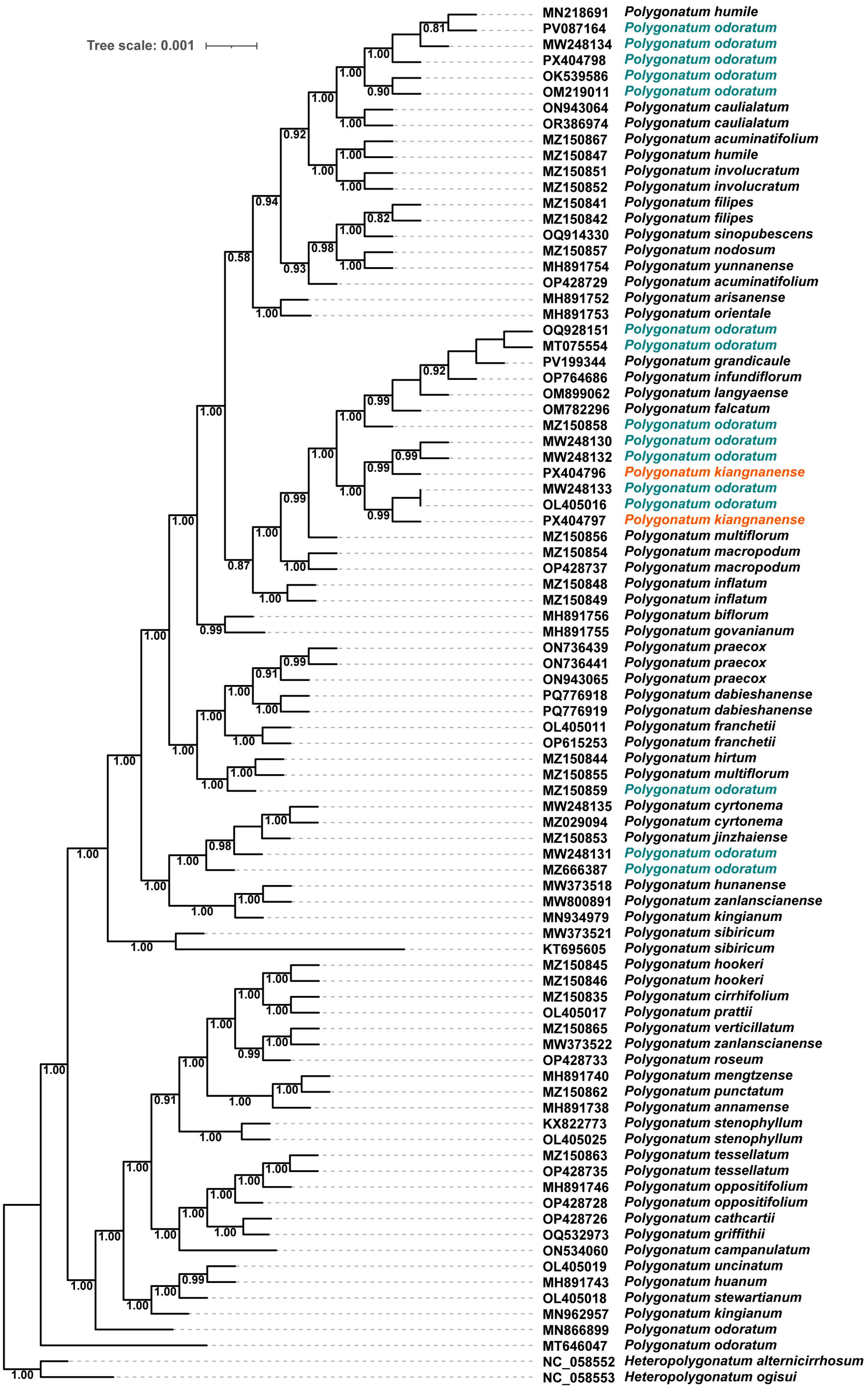

Figure S1 A preliminary phylogenetic tree constructed with representative chloroplast genomes of all reported *Polygonatum* species.
