# Supplementary figures and images for: Polygonatum kiangnanense (Asparagaceae), a new species from southeastern China
Source: PhytoKeys. 2026 Jul 21;277:227–40. doi: 10.3897/phytokeys.277.198454 (PMC13417040; doi:10.3897/phytokeys.277.198454)

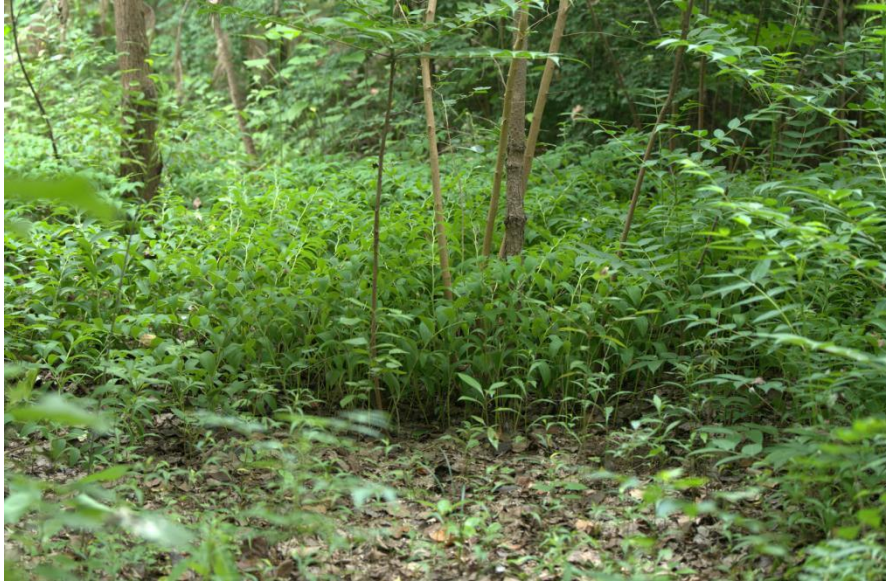

Figure S2 *Polygonatum kiangnanense* habitat.

Supplement: Supplementary material 2 — Polygonatum kiangnanense habitat [file phytokeys-277-227_article-198454__-s002.pdf]
